# Priming food intake with weight control cues: systematic review with a meta-analysis

**DOI:** 10.1186/s12966-018-0698-9

**Published:** 2018-07-09

**Authors:** Nicola J. Buckland, Vanessa Er, Ian Redpath, Kristine Beaulieu

**Affiliations:** 1 Department of Psychology, University of Sheffield, Cathedral Court, 1 Vicar Lane, Sheffield, S1 2LT England; 2 Population Health Sciences, Bristol Medical School, University of Bristol, Canynge Hall, 39 Whatley Road, Bristol, BS8 2PS England; 3 The Behaviouralist Ltd, 5 Hoxton Square, London, N1 6NU England; 4 Appetite Control and Energy Balance Group, School of Psychology, University of Leeds, Leeds, LS2 9JT England

**Keywords:** Food intake, Goal priming, Weight control goals, Systematic review, Meta-analysis

## Abstract

**Background:**

A growing number of studies suggest that exposure to cues which are associated with weight control can prime or prompt controlled food intake in tempting food environments. However, findings are mixed and understanding which types of cues and for whom such cues may be most effective is needed to inform subsequent research and societal applications. A systematic review and meta-analysis were conducted to evaluate the effects of exposure to weight control cues compared with control cues on food intake.

**Methods:**

PsycINFO, Medline, Embase and Web of Science were searched using key terms. Hedge’s g was used to calculate effect sizes based on mean food intake, standard deviations and sample sizes extracted from relevant publications and, a random effects model was used for the meta-analysis.

**Results:**

Twenty-five articles consisting of 26 studies were eligible. Data from 25 studies (31 effect sizes) were available for the meta-analysis. Overall, weight control cues reduced food intake, albeit to a trivial effect (ES: -0.149, 95% CI: -0.271 to − 0.027). Subgroup analyses when studies which induced negative affect were removed showed that for individuals with strong weight control goals the effect was small-to-moderate (ES: -0.440, 95% CI: -0.718 to − 0.163), whereas for individuals with weak weight control goals this effect was trivial and non-significant (ES: 0.014, 95% CI: -0.249 to 0.278). Cue type and level of engagement did not significantly moderate the effect; however, specific cues (low-calorie foods and thin models) and attended engagement yielded significant effects. Caution is needed interpreting these findings as most studies were rated with high risk of bias and a number of studies could not be included in the subgroup analyses.

**Conclusions:**

Based on the data available, weight control cues reduce food intake in individuals with strong weight control goals. Further research is needed to explore longer term effects of cue exposure and confirm underlying mechanisms. PROSPERO registry#CRD42016052396.

**Electronic supplementary material:**

The online version of this article (10.1186/s12966-018-0698-9) contains supplementary material, which is available to authorized users.

## Background

The current obesogenic environment presents constant exposure to palatable high energy dense foods and has been identified as a key driver of overconsumption and rising obesity rates [1, 2]. Exposure to palatable food cues can increase physiological responses to food [3], anticipatory food reward [4] and food intake [5]. As such, it has been recognised that interventions which target environmental cues might offer an effective strategy to influence eating behaviour at the population level [6]. Indeed, there is a growing body of evidence that small alterations to the proximal environment can influence pro-health behaviours [6].

One way that environmental cues can influence behaviour is by activating or priming cognitions [7]. According to goal priming theorists, cues can activate cognitive goals and result in goal-directed behaviour [7]. While the cue exposure can occur at a conscious or subconscious level, the activation of goals occurs outside of conscious awareness. Applied to eating behaviour, this suggests that cues associated with weight control (e.g. scales, low calorie foods) can activate or prime weight control goals and lead to controlled or reduced food intake [8]. According to The Goal Conflict Theory, such effects will be more pronounced in individuals who hold relevant weight control goals [8]. In support, laboratory studies have reported that compared to control cues, exposure to weight control cues have reduced subsequent food intake in general samples [9, 10] and in individuals with strong weight control goals such as restrained eaters [11] and dieters [12, 13]. Other studies have reported that the effects of weight control cues are moderated by particular settings such as the time of day (effects in general sample) [14] and portion size (effects in restrained eaters only) [15]. The effects of weight control cues on food intake (in restrained eaters) have also been found in real world settings such as in response to a ‘slimming poster’ displayed on the entrance to a butcher’s store [16]. These findings are important because they suggest that goal priming can be applied to population-level behaviour change interventions [17].

However, findings are mixed as some studies reported no effects of weight control cues on food intake [18, 19]. Such discrepant findings might be due to the methodologies used across studies as the types of cues used, the level of cue engagement (for example, subliminal, incidental and attended) and the samples tested have largely varied. Understanding the effects of weight control cues and which types of cues, settings and for whom these cues might be most effective will be valuable to inform societal applications and subsequent research.

As such, a systematic review and meta-analysis of the evidence was conducted to identify the effectiveness of weight control cues on food intake and to investigate if the effects are moderated by the type of weight control cue used (cue type and level of engagement) and the extent to which participants hold weight control goals.

## Methods

### Search strategy

The systematic review and meta-analysis is reported in line with the preferred reporting items for systematic reviews and meta-analysis (PRISMA) guidelines. The protocol was registered in the PROSPERO database (International prospective register of systematic reviews; registration number: CRD42016052396). Four electronic databases were searched for articles published up to January 2017 (and the search was updated in March 2018): PsycINFO (from1806), Medline (from 1946), Embase (from 1947) and Web of Science (from 1864). The search included a combination of key words relevant to cues, weight control and food intake (Additional file 1). One author conducted the search and selected articles for full text screening based on article titles and abstracts (NB) and a second author (KB) checked 10% of articles (there were no disagreements). A manual search of eligible articles reference lists and citations was also conducted which identified two eligible articles [20, 21]. Authors of eligible studies were contacted to request for other published or unpublished studies to minimise publication bias. This resulted in two articles [11, 22] and one unpublished study being identified [23].

### Study eligibility criteria

The search was limited to English-language papers, human studies and healthy adults aged 18–64 years. Studies were included if they exposed participants to cues associated with weight control and, during or after cue exposure, objectively measured food intake as either energy intake, weight consumed or piece count. Studies that used self-reported food intake, food choice or eating intentions were not included. Only food intake was assessed as it allows for the precise measurement of consumption [24], whereas food choice does not necessarily reflect intake and self-report measures are subject to underreporting [25]. To our knowledge, there is no formal definition or a database of validated cues that are associated with weight control cues. Therefore, we considered studies that used cues closely linked with dieting constructs (e.g. slim models, weighing scales, low calorie foods, weight management products and exercise-related cues) to be eligible. There were no disagreements between authors about whether a particular cue was regarded as a weight control cue or not. Cues more closely aligned to eating enjoyment cues (e.g. overweight body images, palatable food) were not considered to be weight control cues. Studies were included regardless of the theoretical approach used (e.g. some studies used exposure to slim models to manipulate negative body image, body dissatisfaction or motor priming [26] rather than priming weight control goals per se [20–22, 27–30]). Exposure to cues could be either subliminal, incidental or explicit. Studies which administered cues after at least 5 min of access to snacks were excluded as this could have minimised the impact of cue exposure on food intake.^1^ Studies using food packaging labels as cues (e.g. ‘low fat’) were excluded to prevent any confusion over inconsistent food messages confounding food intake (e.g. ‘healthy’ cookies). Studies that had cues which incorporated negative messages about being overweight were excluded (e.g. body weight stigma) [31]. To prevent study duplications, PhD and Masters theses containing studies published in peer-reviewed journal articles were not included. Experimental, quasi-experimental and intervention studies which used either within- or between-subject designs were included. For quality control, only studies that included a control condition comprising of either no cue or a neutral cue were included; studies that compared weight control cues to eating enjoyment cues only (aimed at increasing food intake) and did not include a control condition were excluded. Two authors were responsible for screening full text articles (NB, KB). There were no disagreements.

### Data extraction

One author extracted sample sizes, means and standard deviations of food intake in the cue and control conditions (KB). Another author (NB) checked that the extracted data corresponded with the data reported in papers (NB). One author (NB) extracted all other study information. The extracted data is shown in Table 1. Authors were contacted for missing sample sizes, means, standard deviations and units of outcome (grams or kcal). In instances when means and standards errors were provided for food intake, standard deviations were calculated [10, 21].

**Table 1:** Description of data collected from included articles

Criterion	Data extracted
Country research conducted	Country
Study design	Between-subjects, within-subject, laboratory, field.
Participant characteristics	Total sample size; number of male and female participants; mean, median, standard deviation and range for age and BMI and BMI assessment method if assessed (self-report or objectively measured).
Moderating variables	Individual differences in eating behaviour traits [dieting status; restrained eating (scale used) or any other psychometric scales]; any other moderators examined.
Cue type and level of engagement	Type of cue: Specific item (e.g. slim models, foods); level of engagement: explicit, incidental, sub-conscious; cue validation
Test food	Interval between cue exposure and assessment of food intake; Test foods used: snack, meal, sweet, savoury, food name.
Mechanism for effects tested?	Yes, no; type of assessment used.
Main outcome	Food intake in ounces, piece count, grams and energy intake; assessment method for food intake (weighed, piece count)
Risk of bias	Random allocation to conditions, randomisation methods, allocation concealment, blinding (use of a cover story and participants' beliefs about the study aims; whether the researcher was aware of the study aims or condition that had been administered), completion of outcome reporting (excluded participants), procedures used to control for appetite, individual or social setting, administration of psychometric scales.

### Meta-analysis

A specialty meta-analysis software was used for the analyses (Comprehensive Meta Analysis, version 3; Biostat, Englewood, NJ). Means, standard deviations and sample size for the cue exposure and control conditions were inputted into the software. For studies with multiple comparisons (e.g. [32]), sample sizes for each comparison were adjusted accordingly. In one study [19], means, sample size and *p*-value were used to compute the effect size (ES). The ES was calculated as Hedges’s g to account for potential bias and the overall ES using a random effects model due to large variability in study designs and outcomes reported. A negative effect size value indicates that cue exposure decreased food intake whereas a positive effect size indicates that cue exposure increased food intake relative to no cue exposure. The effect sizes were interpreted as follows: < 0.2 as trivial, 0.2–0.3 as small, 0.5 as moderate, and > 0.8 as large [33]. Heterogeneity was assessed using the I^2^ index, with values of 25% considered as low heterogeneity, 50% as moderate and 75% as high [34]. Sensitivity analyses were conducted by the software by excluding one study at a time to examine if results were affected by any one study in particular. Planned subgroup analyses were conducted to identify whether the effects of weight control cues on food intake was moderated by cue type, level of engagement and whether participants held weak or strong weight control goals. High restrained eaters, dieters and individuals with high self-discrepancy were combined and classified as those with strong weight control goals, and low restrained eaters, non-dieters and individuals with low self-discrepancy were classified as those with weak weight control goals (using restrained eating as an indicator for weight control goals is consistent with previous research [8]) (the decision to combine dieters, restrained eaters and self-discrepancy was made after data extraction and not pre-specified in the registered protocol). Exploratory moderator analyses were also conducted for categorical data, including sex, snack type, sample type, intake measure, use of appetite control procedures and theoretical model. To assess publication bias, Egger’s regression [35] and the trim-and-fill method were used [36].

### Risk of bias

Risk of bias was assessed using the Cochrane Collaboration’s tool as closely as possible [37] (Additional file 2). Studies were evaluated for ‘blinding of participants and personnel’ and ‘blinding of outcome assessors’ based on the likelihood that participants were naive about food intake being assessed (i.e. use of a cover story and whether the cover story was believed), and whether the experimenter was blinded to the study aims or condition that had been administered. For ‘other bias,’ studies were assessed based on the likelihood that confounding variables could have influenced food intake [for example piece count (high risk of researcher bias), the absence of procedures to control for appetite between conditions [38]; social settings [24, 39]; providing restricted food portions [24]; and administering psychometric scales prior to the assessment of food intake which may have increased body image awareness]. One author rated each study for risk of bias (NB) and decisions were cross-checked by another author (KB). Any disagreements were discussed and resolved between the two authors.

## Results

### Included studies

**Fig. 1:**
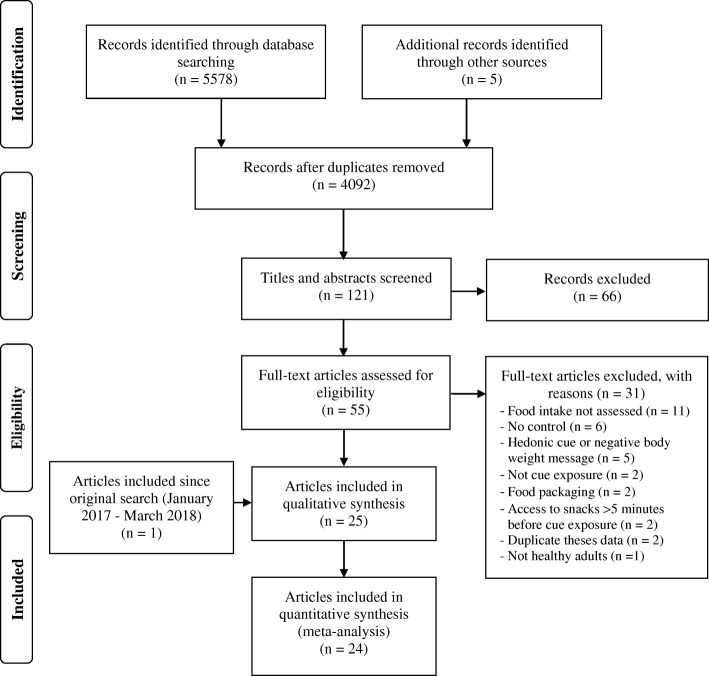
Flow chart of the study selection process

Figure 1 shows the article selection process. Of the 5583 articles identified, 25 were eligible for the systematic review which comprised of 26 studies. Of these, one article was excluded from the meta-analysis as the data (means, standard deviations and sample sizes) could not be obtained [28]. As such, the meta-analysis included 24 articles from which there were 25 studies (one article had two studies [26]) with 31 relevant comparisons (four studies included two comparisons [10, 14, 15, 40] and one included three [32]). For one study, different cues were used for males and females and only the cue used for females met the eligibility criteria [32]. Therefore, for that study, only the data for females were included in the systematic review and meta-analysis.

### Study designs and participants

Table 2 displays characteristics of the eligible studies. Of the 26 studies, one was a field study [16] and all others were laboratory studies. All used between-subject designs except for two which used within-subject designs [13, 23]. Eight studies were conducted in the USA [14, 19, 26, 32, 40–42], four in the UK [12, 13, 20, 23], three in Canada [18, 21, 30], three in the Netherlands [15, 16, 27], three in Switzerland [9, 11, 43] two in Australia [10, 29], two in New Zealand [22, 28] and one in France [44].

**Table 2:** Characteristics of articles eligible for review

First author (year), Country	Participants and design^a^	Cue type and level of engagement	Test food, timing and outcome	Moderating variables (assessment method)	Main result for food intake	Notes and methodological considerations
Albarracin (2009); Study 1; US [26]	Laboratory; between-subjects; random assignment to conditions; Student sample, *n* = 53 (72% males) [*n* = 26 in prime condition (assumed)]; Age: 18.90 (1.11); BMI: 22.20 (3.85) (assessment method not reported); No exclusions	Five print advertisements (duration: not specified) Experimental: exercise campaign adverts with action words (‘go for a walk’; ‘join a gym’; ‘Go skating’ Control: general adverts with non-exercise messages: ‘make friends;’ ‘be in a group;’ ‘be together’ Attended (participants rated appeal and efficacy of adverts) No cue validation	Raisins (*n* = 20) (sweet) Timing: immediately after cue exposure Outcome: EI (assessed with piece count)	None	EI was greater in the experimental compared to control condition	No procedures to control for appetite between conditions; Cover story used. No info about participants’ beliefs about the aims of the study; Small portion size might have constrained food intake No test of mechanism
Albarracin (2009); Study 2; US [26]	Laboratory; between-subjects; random assignment to conditions; Student sample; *n* = 51 (46% males); [*n* = 25 in prime condition (assumed)]; Age: 19.26 (1.16); BMI: 22.80 (4.25) (assessment method not reported); No exclusions	Eight words (duration: not specified) Experimental: action words (active, go); Control: neutral words ‘pear;’ ‘moon;’ Subliminal (15 milliseconds) No cue validation	Raisins (sweet), M&Ms. (sweet, peanuts (savoury) (15 of each) Timing: immediately after cue exposure Outcome: EI reported (assessed with piece count)	None	EI was greater in the experimental compared to control condition	No procedures to control for appetite between conditions; Cover story used; No info about participants’ beliefs about the aims of the study; Small portion size might have constrained food intake No test of mechanism
Boland (2013); Study 2^b^; US [14]	Laboratory; between-subjects; random assignment to conditions; Student sample, *n* = 149 (46.1% males) in morning [*n* = 37 in prime and control (assumed)] and afternoon sessions [*n* = 37 in prime and 38 in control conditions (assumed); Age: 20.17; BMI not reported; No exclusions	Word search puzzle with thirteen words (duration: not specified) Experimental: Included seven ‘healthy’ words (energetic, exercise, fitness, healthy, nutritious, strong and thin); Control: included seven neutral words (alligator, gasoline, magazine, mountain, picture, ranch, shampoo) Attended (searched for words) No cue validation	M&Ms. (sweet) Timing: immediately after cue exposure and while watching a television programme Outcome: Ounces (weighed intake)	Time of day: morning, afternoon.	No main effect of condition. Significant condition x time of day interaction, food intake was significantly lower in the experimental condition compared to control in the afternoon. Food intake did not differ between conditions in the morning session	Not random allocation to morning or afternoon sessions; No procedures to control for appetite between conditions (pre-test showed no difference between morning and afternoon, no data reported comparing appetite between experimental and control conditions); Not clear info about cover story; No participants guessed study purpose; SD for age not provided, range: 18–27 years. No test of mechanism
Bourn (2015); AU [29]	Laboratory; between-subjects; random assignment to conditions; Student sample, *n* = 99 females (*n* = 48 in prime condition); Age: 19.35 (2.11); BMI: 23.51 (5.19) (self-reported); No exclusions	Reality television programme (duration: 10 min) Experimental: ‘The Biggest Loser’ focused on weight loss and appearance of four female contestants; Control: ‘The Block’- renovated apartment Attended No cue validation	Chocolate (sweet), corn chips (savoury) and mixed dried fruit (sweet) Timing: during cue exposure. Outcome: EI (weighed)	None for food intake	No significant differences in food intake between conditions	No procedures to control for appetite between conditions; Cover story used; No info about participants’ beliefs about the aims of the study; Of the 99 participants, 79 did not consume any test foods. No test of mechanism
Boyce (2013); NZ [22]	Laboratory; between-subjects; random assignment to conditions; Student sample, *n* = 100 females [*n* = 50 in prime condition (assumed)]; Age: 21.92 (3.90); BMI: 22.89 (3.18) (measured by researcher); Exclusions *n* = 46 (*n* = 29 excluded for guessing study aims; *n* = 7 who were obese; *n* = 7 with food allergies; *n* = 3 who were middle aged)	Seven magazine advertisements (slideshow) (duration: each slide displayed for 20 s) Experimental: Five featuring thin and attractive models (product based: (perfume or make up) and two filler adverts .Control: Same as experimental but with thin models removed. Attended (participants were asked to take in as much detail as possible) No cue validation	Chocolate (sweet) and crispy M&Ms. (sweet) Timing: after cue exposure participants completed a memory task and an implicit mood task (with positive or negative emotional words) and were then provided with snacks. Outcome: Grams (weighed)	Restraint: continuous (Restraint Scale, Concern for Dieting Subscale only and Dietary Intent Scale) [57]	No significant differences in food intake between conditions. Intake did not differ at varying levels of restraint across conditions	No procedures to control for appetite between conditions however pre-study hunger (7-point scale) was included as a covariate in analyses; Cover story used and participants who guessed study aims were excluded; Administering the implicit mood and weight satisfaction task prior to the snack test might have primed positive or negative emotions or negative body image; Insufficient data available to be included in meta-analysis Tested implicit mood as a potential mechanism
Boyce (2014); NZ [28]	Laboratory; between-subjects; random assignment to explicit (*n* = 96) and incidental (*n* = 78) conditions and then random assignment to prime (explicit *n* = 47; incidental *n* = 39) and control conditions; Student sample, *n* = 174 females; Age: 20.43 (6.29); BMI: 23.06 (2.75) (measured by researcher); Exclusions *n* = 75 (*n* = 29 for guessing study aims; *n* = 26 with BMI ≥ 30; *n* = 20 did not engage with explicit images).	Seven images (duration: two-minute slideshow, each slide shown for 20 s); Experimental: featured thin women advertising beauty products; Control: seven neutral images (e.g. furniture) Attended (told to concentrate on images for a subsequent memory test) and incidental (images were shown in an adjacent room while participants completed a coping style questionnaires). No cue validation	Pretzels (savoury), savoury crackers, chocolate/peanut M&Ms. (sweet), bite-sized cookies (sweet) Timing: after completing a lexical decision task and rating hunger Outcome: Grams (weighed)	Restraint: continuous (Restraint Scale, Concern for Dieting subscale only and Dietary Intent Scale) [57] Level of engagement: explicit versus incidental	Restraint positively correlated with sweet food intake in the explicit experimental condition; No significant associations in the incidental or control conditions; Savoury food intake was lower in the incidental prime condition compared to control. Intake did not differ in the explicit condition or at varying levels of restraint.	No procedures to control for appetite between conditions; post-cue hunger was included as a covariate in savoury food analyses (not sweet); Cover story used and participants who guessed the study aims were excluded; In the inadvertent condition, completing a questionnaire on coping styles might have primed confounding constructs; Insufficient data available to be included in meta-analysis No test of mechanism
Brunner (2012); Study 1; CH [9]	Laboratory; between-subjects; Community sample; *n* = 95 (30% males) (*n* = 47 in prime condition); Age: 35.4; BMI not reported; 1 outlier excluded (food intake > 3 SD)	Picture on laptop computer screensaver (duration: 5 min) Experimental: three thin human-like sculptures from Piazza by Giacometti; Control: Orange and yellow painting by Rothko Incidental (‘unobtrusively’ displayed on a computer next to participant) No cue validation	Chocolate pieces (*n* = 20) (sweet) Timing: during cue exposure Outcome: Piece count	None	Food intake was lower in the experimental compared to control condition	Not specified if random allocation to conditions; No procedures to control for appetite between conditions; hunger (time point collected not reported) was included as a covariate; Cover story used. No info about participants’ beliefs about the aims of the study; SD for age not provided, range: 16–74 years. No test of mechanism
Buckland (2013)^c^; UK [13]	Laboratory; within-subjects; randomised order of conditions; Mixed community and student sample; *n* = 26 females; Age: 30.03 (9.28); BMI: 25.50 (4.56) (measured by researcher); Exclusions *n* = 13 (*n* = 6 excluded for guessing study aims; *n* = 6 dieting to maintain weight; *n* = 1 inconsistent lunch times)	Consumption of a preload before evening meal (284 g) (duration: 10 min) Experimental: salad (100 kcal); Control: Water Attended (listed first thoughts associated with preload, reported frequency of consumption and reported a memory associated with salad) Cue validation: pre-study online survey (*n* = 230) showed the priming cue (salad) was associated with dieting to lose weight	Evening meal: cheese and tomato pizza Timing: after cue exposure participants completed appetite and mood ratings, a lexical decision task and were then provided with test meal Outcome: EI (weighed)	Dieting status: dieting to lose weight, not dieting Restraint (R) and disinhibited (D) eating styles: HRHD, HRLD, LRHD, LRLD [Three Factor Eating Questionnaire (TFEQ) [58]	Energy intake was lower in experimental condition compared to control. A significant condition x diet status interaction showed non-dieters’ EI did not differ between conditions, dieters consumed significantly less in experimental compared to control	Control procedures used concerning alcohol intake evening before test session, similar physical activity across test days, fast two hours prior to lunch; fixed lunch provided four hours before evening meal; Cover story used and participants who guessed the study aims were excluded; Mechanism tested: A lexical decision task assessed goal accessibility (using 15 diet-related words) after cue exposure
Buckland (2014); UK [12]	Laboratory; between-subjects; random assignment to conditions; Mixed community and student sample, *n* = 67 females (*n* = 35 in prime condition); Age: 23.67 (5.87); BMI: 23.48 (2.88) (measured by researcher); Exclusions n = 2 (*n* = 1 food intake outlier; *n* = 1 BMI outlier)	Nine subliminal images (duration: 23 milliseconds each; two exposure phases) Experimental: low calorie foods and beverage items (fruits and diet products); Control: non-food objects Subliminal Cue validation: participants (*n* = 55) rated priming cues as being more associated with losing weight compared to control cues	Four snacks – a low and high fat sweet and savoury snack Timing: after second cue exposure phase participants completed appetite and mood ratings and were then provided with snacks Outcome: EI (weighed)	Dieting status: dieting to lose weight or maintain weight, not dieting Restraint and disinhibited eating styles (TFEQ [58])	No main effect of condition. Condition x diet status interaction approached significance; non-dieters’ EI did not differ between conditions; dieters consumed significantly less in experimental condition compared to dieters in control (same results for high restrained high disinhibited eaters)	Control procedures: instructed to fast two hours prior to a fixed lunch and fast between lunch and test session; Cover story used. Inclusion of participants who guessed the study aims did not affect the results; Mechanism tested: A lexical decision task assessed goal accessibility (using 4 diet-related words) after cue exposure
Buckland (unpublished); UK [23]	Laboratory; within-subjects; randomised order of conditions; Mixed community and student sample, *n* = 30 females; Age: 27.67 (11.54); BMI: 24.92 (4.01) (measured by researcher); Exclusions *n* = 16 (*n* = 14 dieting to maintain weight; *n* = 2 methodological issues)	Exposure to the sight and smell of an object (duration: 10 min and remained during snack test) Experimental: a fresh orange Control: non-food object (soap) Attended (participants were instructed to intensely smell the cue three times) Cue validation: pre-study online survey (*n* = 180) showed the priming cue (orange) was associated with dieting to lose weight	Cheesy bite crackers (savoury), toffee popcorn (sweet), chocolate chip cookies (sweet) and salted crisps (savoury) Timing: after cue exposure participants completed appetite and mood ratings, a lexical decision task and were then provided with snacks Outcome: EI (weighed)	Dieting status: dieting to lose weight, not dieting Restraint (R) and disinhibited (D) eating styles: HRHD, LRHD, LRLD (TFEQ [58])	No main effects and no significant condition x diet status interactions on food intake	Control procedures: Fixed lunch provided two hours prior to test session. Instructed to fast two hours between lunch and test session; Cover story used. Inclusion of participants who guessed the study aims did not affect the results.; Mechanism tested: A lexical decision task assessed goal accessibility (using 15 diet-related words) after cue exposure
Harris (2009)^d^; Study 2; US [19]	Laboratory; between-subjects; random assignment to conditions; Student sample, *n* = 64 (assumed even split across three conditions) (32% males) [*n* = 32 in prime condition (assumed)]; Mean age and BMI not reported Exclusions: *n* = 4 for guessing study aims	Eleven television commercials during a 16 min comedy television programme (total 11 min including filler commercials) Experimental: Four featured food and beverages which included a nutrition message (granola bar, orange juice, oatmeal and an instant breakfast beverage; Control: All non-food commercials Attended Cue validation: Pre-study (*n* = 55) showed the priming cue was rated as being more associated with nutrition and health compared to ‘snack indulgent’ commercials (no comparison to control)	Carrots and celery with dip (savoury); mini chocolate chip cookies (sweet) and cheesy snack mix (savoury), trail mix (savoury) and multi-grain tortilla chips (savoury) Timing: After cue exposure, participants completed a mood assessment and rated hunger and thirst and were then provided with snacks Outcome: Grams (weighed)	Restraint: low, high (Restraint Scale [57]) Sex: males, females	No significant differences between food intake in the experimental and control condition	No procedures to control for appetite between conditions; hunger at pre- and post-cue exposure was included in analyses; Cover story used and participants who guessed the study aims were excluded; Age range: 18–24 years. Means and SDs were obtained from figures Means and SDs were not available for sex comparisons or restraint (only z-scores) Tested hunger and mood as potential mediators, no tests of goal priming as a mechanism
Harrison (2006)^e^ US [32]	Laboratory; between-subjects; random assignment (one of four conditions: control, image only, image with congruent or incongruent text); Student sample, *n* = 222 females (control *n* = 40; image only *n* = 54; image/congruent text *n* = 67); Age: 19.62 (1.12); BMI: 22.68 (4.40) (self-reported); No exclusions	Thirty images displayed on a slideshow (duration: each image shown for 30 s); Experimental: attractive young women. Three conditions: images alone or images shown with congruent (motivational language to become thinner or more toned) or incongruent text [about Aruba] Control: no images (one condition) Attended (participants rated appeal of each image) Pre-study sample (*n* = 12) rated priming cues as representative of thin ideals	Full size pretzels (savoury) Timing: After cue exposure participants completed a questionnaire with self-concept measures and were then provided with snacks Outcome: Piece count	Self-discrepancy between perceptions of actual body and perceptions of what peers thought they ought to have: low, high (Self-Discrepancy Questionnaire [59])	Intake did not differ between conditions for women with low discrepancy. Women with high discrepancy consumed less in experimental conditions (image and image with congruent text) compared to control (image with incongruent text did not differ to control)	No control procedures for appetite between conditions; No info about cover story or participants’ beliefs about study aims; Food intake was assessed in groups of same sex peers (*n* = 3–9) (risk confounded by social norms); researcher initiated food intake and then recorded participants’ intake (researcher bias); Three experimental conditions were compared to one control condition; sample sizes for each comparison were adjusted accordingly. No tests of mechanism
Jansen^f^ (2002); NL [27]	Laboratory; between-subjects; random assignment to conditions; Student sample, *n* = 36 females (*n* = 19 in prime condition); Age: 19.8 (1.6) BMI: 23.01 (3.1) (measured by researcher; Exclusions *n* = 4 (*n* = 3 for guessing study aims; n = 1 food intake outlier)	Eight images on slideshow (duration: each displayed for 15 ms) Experimental: thin media models; Control: neutral (office equipment – stapler, pencil, telephone) Subliminal (15 ms) Cue validation: pre-study sample (*n* = 4) rated the priming cues as representing thin ideal body images	High calorie individually chosen snack Timing: After cue exposure participants rated mood, self-esteem and completed an image awareness task and were then provided with snacks Outcome: EI (weighed)	Restraint: low, high (Restraint Scale [57])	No main effects of condition and no significant condition x restraint interaction on EI	Control procedures: instructed to eat a small meal and then fast for 2 h prior to test session; Cover story used and participants who guessed the study aims were excluded; Administering the self-esteem scale prior to the snack test might have primed thoughts about self-esteem Details about the specific test foods provided was not available Mechanisms tested: mood and self-esteem were assessed as mechanisms. No tests for goal priming
Mills (2002) Study 1^g^; CA [21]	Laboratory; between-subjects; random assignment to conditions; Student sample, *n* = 73 females (*n* = 28 in prime condition); Age: 19.72 (1.13); BMI: 23.78 (self-reported; mean of restrained and unrestrained eaters’ BMI) No exclusions	Twelve laminated magazine adverts (duration: 15 min) Experimental: Seven with full-body thin and attractive female models and five filler adverts; Control: all product only adverts Attended (participants rated adverts on multiple attributes) Cue validation: pre-study team of researchers rated images as thin and attractive	Three different flavoured cookies (sweet) Timing: after cue exposure participants completed mood, self-esteem, and body-size perception and were then provided with snacks Outcome: grams (weighed)	Restraint: low, high (Restraint Scale [57])	Significant condition x restraint interaction: unrestrained eaters’ intake did not differ between conditions, restrained eaters ate more in the experimental compared to control condition	No control procedures for appetite between conditions; Cover story used, no participants guessed study aims; Administering the self-esteem scale prior to the snack test might have primed thoughts about self-esteem BMI SD not available Mood and body image assessed as potential mechanisms. No tests for goal priming
Minas (2016); US [41]	Laboratory; between-subjects; random assignment to conditions; Student sample, *n* = 161 (51.6% males) (*n* = 82 in prime condition); Age: 19.9; BMI not reported No exclusions	Scrambled sentence computer game task (duration: 8 min) Experimental: included one or two body image words (slim, fit, weight, diet, healthy, slender); Control: neutral words (worker, room, leaves, bench, dirt, dwell) Attended (participants created a five word headline from a set of provided words) No cue validation	Baked or regular Ruffles crisps (savoury) Timing: After cue exposure participants completed a demographic survey, rated the cue exposure game and were then provided with snacks while watching a television programme Outcome: EI (weighed)	Restraint: continuous (Restraint Scale [57]) Sex: males, females	No main effects of condition. Significant condition x sex interaction: males’ intake did not differ between condition; females’ EI was less in experimental compared to control	No control procedures for appetite between conditions; Cover story used; no info about participants’ beliefs about the study; SD for age not provided; Means and SD unavailable for restraint analyses No tests of mechanism
Papies (2010); NL [16]	Field (butcher’s store); between-subjects; Community sample, *n* = 156 (43.6% males) [*n* = 76 in prime condition; Age: 56 (14.18); BMI: 26.50 (4.45) (self-reported); No exclusions	Poster on entrance to a Butcher’s store (duration: N/A) Experimental: Announced a recipe ‘good for a slim figure and low in calories;’ Control: no poster Incidental (attention was not explicitly directed to the poster) No cue validation	Meat snacks (e.g. meatballs) (savoury) Timing: not reported (differed for each participant) Outcome: Piece count	Restraint: low, high (Restraint Scale, Concern for Dieting subscale only [57]) Sex: males, females	Significant condition x restraint interaction: unrestrained eaters’ intake did not differ between conditions; restrained eaters consumed less in the experimental compared to control condition	Not random assignment to conditions; No control procedures for appetite between conditions; No cover story used, given subtle nature of manipulation unlikely participants guessed the aim; Of the 156 participants, 86 consumed no snacks; Means and SDs used in meta-analysis were for males’ and females’ intake obtained from author For subgroup restraint analysis, means, SDs and sample sizes were estimated No tests of mechanism
Pelaez-Fernandez (2011)^h^; CA [18]	Laboratory; between-subjects; random assignment to conditions; Student sample, *n* = 97 females (*n* = 49 in prime condition); Age: 19.95 (3.75); BMI not reported; No exclusions	Magazine covers placed on table while participants completed consent forms (duration: 10 min) Experimental: fit females/ slim models; Control: Geographic scenes and furniture Incidental (attention not diverted to magazines) No cue validation	Three types of cookies (sweet) Timing: after cue exposure half of participants completed a goal accessibility task and were then provided with snacks, the other half were provided with snacks immediately after cue exposure and then completed the goal task Outcome: Grams (author confirmed in correspondence) (weighed)	Restraint: low, high (Restraint Scale [57])	No main effects of condition and no significant condition x restraint interaction on food intake	No control procedures for appetite between conditions; Cover story used, no info about participants’ beliefs about the study Mechanism tested: A goal accessibility task (using 8 diet-related words) was completed after cue exposure or after the snack test (order was counterbalanced across participants)
Seddon & Berry (1996); UK [20]	Laboratory; between-subjects; random assignment to conditions; Mixed community and student sample, *n* = 74 females (*n* = 37 in prime condition); Age: 25.6 (7.7); BMI not reported; No exclusions	Television commercials (duration: 12 min) Experimental: featuring thin and attractive females; Control: neutral, no thin and attractive women Attended (participants informed they would be asked questions about the adverts after viewing them) No cue validation	Salted peanuts (savoury), chocolate coated peanuts (sweet), pickled onion savoury snack (savoury) Timing: After cue exposure, participants completed a self-esteem measure and were then provided with snacks Outcome: Grams (weighed)	Restraint: low, high (Restraint Scale [57])	Significant condition x restraint interaction; post hoc tests showed no significant differences between conditions at varying levels of restraint (restrained consumed more than unrestrained in prime condition, no differences in control)	Control procedure: two hour fast prior to test session; Cover story used, no info about participants’ beliefs about study Administering the self-esteem scale prior to the snack test might have primed thoughts about self-esteem Mechanism tested: self-esteem assessed
Sellahewa (2015); AU [10]	Laboratory; between-subjects; random assignment to self-control depletion/no depletion and priming/no priming (2 × 2 design); Student sample, *n* = 85 (25.9% males) [21 in each except for *n* = 22 in non-depletion/goal priming (prior to exclusions)] (25.9% males); Age: 20.08 (3.96); BMI: 21.37 (2.65) (self-reported); Exclusions *n* = 6 (n = 4 for guessing study aims; *n* = 2 non-compliant)	Scrambled sentence computer task (duration: determined by participant) Experimental: nine health-related words (active, exercise, fit, vigorous, healthy, sunscreen, well, wellbeing, wholesome); Control: nine neutral words (calm, clever, deodorant, generous, logical, practise, rad, sassiness, spiritual) (words were obtained from authors) Attended (participants rearranged order of sentence to form a grammatically correct sentence) No cue validation	Chocolates (sweet), savoury biscuits, potato chips (savoury) Timing: Immediately after cue exposure Outcome: Grams (weighed)	Levels of self-control: self-control, self-control depletion [achieved by allowing (self-control) or suppressing (self-control depletion) emotional response to humorous video]	Participants consumed significantly less in the experimental compared to control condition. Condition x depletion interaction on food intake was non-significant.	No procedures to control for appetite between conditions; hunger was included as a covariate; Cover story used and participants who guessed the study aims were excluded; Only one example of the healthy words were provided. No test of mechanism
Stampfli (2016); CH [43]	Laboratory; between-subjects; four conditions – low/high cognitive load and priming/no priming (2 × 2 design; Community sample, *n* = 128 (26.6% males) [*n* = 62 in prime condition (obtained by correspondence with author)]; Age: 46.35 (14.20); BMI not reported; 9 excluded for not fulfilling requirements of cognitive load task)	Picture on computer screensaver (duration: approximately 30 s) Experimental: three thin human-like sculptures by Giacometti moving on a black background; Control: static white picture Incidental (screensaver displayed as participants entered room and chose a cubicle and seated themselves) No cue validation	Crisps (n = 20) (savoury) Timing: after cue exposure, participants completed a cognitive load task and were then provided with snacks Outcome: Grams (weighed)	Cognitive load: low (memorise 2 digits), high (memorise 10 digits) cognitive load. Liking for snack food: low, high snack liking	Food intake was significantly lower in experimental condition compared to control regardless of cognitive load. The effect of condition was only found in participants with high snack food liking, not those with low snack food liking	Not random allocation to conditions; No procedures to control for appetite between conditions; Cover story used, no info about participants’ beliefs about study No test of mechanism
Stampfli (2017); Study 1; CH [11]	Laboratory; between-subjects; four conditions – healthy/unhealthy food, priming/no priming (2 × 2 design); Mixed community and student sample, *n* = 114 (38.1% males) [*n* = 34 in priming unhealthy food condition; *n* = 30 in priming healthy food condition; *n* = 26 in control unhealthy food) (correspondence with author)]; Age: 31.72 (14.11); BMI not reported; Exclusions *n* = 19 (*n* = 18 for guessing study aims and n = 1 for not answering question about study aims)	Picture on laptop computer screensaver (duration: 30 s) Experimental: three thin human-like sculptures by Giacometti moving on a black background; Control: no cue (laptop computer closed) Incidental (screensaver was running while participants entered room and when participants received study instructions (30 s) No cue validation	20 chocolates (sweet) or 20 blueberries (sweet) Timing: Immediately after cue exposure Outcome: Grams (weighed)	Restraint: low, high (German version of the Restraint Scale, Concern for Dieting subscale [60]) Healthiness of food: healthy (blueberries), unhealthy (chocolate)	Food intake was significantly lower in the experimental condition compared to control regardless of snack food healthiness. The effect of condition was only found in high restrained eaters, not unrestrained eaters.	Not specified if random allocation to conditions; No procedures to control for appetite between conditions; Cover story used and participants who had heard about the study before (and thus were aware of the study aims) were excluded; No test of mechanism
Stein (2016); US [40]	Laboratory; between-subjects; randomised to one of four conditions – self-control/self-control fatigue, priming/no priming (2 × 2 design); Student sample, *n* = 84 (34.5% males) (*n* = 20 in self-control/priming; *n* = 22 in no self-control/control; *n* = 21 in self-control fatigue/priming condition); Age: 18.6 (1.0); BMI: 23.03 (3.85) (self-reported); No exclusions for food intake data (analyses with exercise levels *n* = 11 excluded for missing data)	Exercise posters (duration: 20 min) Experimental: Three posters - a man running in the mountains; a photo of the London Olympics with athletes competing in sports; black and white silhouettes of men and women running; Control: neutral artwork on walls Attended (researcher delivered script about the posters to ensure participants noticed them) No cue validation	Cookies (sweet), chocolate (sweet), potato chips (savoury) Timing: 20 min after initial cue exposure (participants completed a self-control or placebo task during cue and snack test interval) Outcome: EI (weighed)	Restraint: continuous (Restraint Scale [57]) Self-control: self-control, self-control fatigue [61] BMI: low, high Tendency towards compensatory eating in response to physical activity: continuous Exercise levels: low, high exercisers	No main effect of condition or self-control depletion on food intake. High exercisers consumed significantly less in experimental compared to control, low exercisers’ intake did not differ between conditions (*n* = 73). All other two-way condition x moderator interactions on food intake were non-significant.	No procedures to control for appetite between conditions; hours since last ate was included as a covariate in the analyses; Cover story used, no info about participants’ beliefs about study; Age SD computed from SEM; No test of mechanism
Strahan (2007); Study 1; CA [30]	Laboratory; between-subjects; random assignment to conditions; Student sample, n = 26 females [*n* = 13 in prime condition (assumed)]; Age range 18–21; BMI not reported; No exclusions	Television commercials (duration: not specified) Experimental: total six – four contained no people and two featured slim models (Victoria Secret commercial with a supermodel, Dove soap commercial with slim woman); Control: total four – none contained people (cellular phone, gas station, pharmacy and insurance company) Attended (participants given goal to remember as much detail as possible about the commercials) No cue validation	Popcorn, whole-wheat crackers, crackers (Ritz) and pretzels (all savoury) Timing: Immediately after cue exposure Outcome: Grams (weighed)	None	Food intake was lower in the experimental condition compared to control	Control procedures: fast three hours prior to test session; Cover story used, no participants guessed the study aims; Means for age not available; No test of mechanism
van Kleef (2011); US [42]	Laboratory; between-subjects; random assignment to conditions; Student sample, *n* = 125 (43.2% males) (*n* = 67 in prime condition); Age: 20.5 (5.0); BMI: 22.7 (3.3) (self-reported); Exclusions: *n* = 3 [n = 2 vegetarians; *n* = 1 outlier for reported levels of physical activity].	Eight television commercials (duration: see condition descriptions) Experimental: exercise equipment and services (running shoes, fitness centre, fitness program) (displayed for 4 min 57 s); Control: did not refer to food or exercise (car insurance, home appliance, pet dog adoption program) (4 min and 59 s) Attended (participants rated commercials and preferences for favourite commercial) Cue validation: Priming cues were rated by participants as making them feel more healthy and in shape compared to ratings of control cues	Lunch meal: pasta dish with tomato sauce, salad and chocolate pudding (salad dressing, cheese and drinks were also available but intake was not recorded) Timing: Immediately after cue exposure Outcome: EI (weighed)	Restraint: low, high (Restraint Scale [57]) BMI: low, high Exercise levels: low, high Exercise intentions: low, high	EI was lower in the experimental condition compared to control. The effect of condition on EI was only found in participants with a high BMI, participants’ EI with a low BMI did not differ between conditions.All other condition x moderators interactions on EI were non-significant.	No procedures to control for appetite between conditions; time since last ate did not differ between conditions; Cover story used, no information about participants’ beliefs about study; No tests of mechanisms
Versluis (2016)^i^; Study 2; NL [15]	Laboratory; between-subjects; random assignment to one of four conditions – small/large portion, priming/no priming (2 × 2 design); Student sample, *n* = 224 (59% males) [*n* = 47 in priming small portion condition; *n* = 55 in priming large condition; *n* = 66 in control small condition]; Age: 21 (1.6); BMI: 25.61 (5.12) (based on mean of BMI across four cells) (self-reported); Exclusions *n* = 34 (*n* = 19 for guessing study aims; *n* = 15 due to allergies or diseases)	Four television commercials inserted in a movie clip (duration: two minutes, 30 s) Experimental: featured ‘healthy’ foods or services (Dannon Light & Fit yoghurt, Weight Watchers, Nike Basketball and Special K breakfast cereal) with messages about resisting tempting foods, dieting and weight loss; Control: non-diet-related (garden furniture, Intel, Phillips Ambilight, Jeep Renegade, Amazon Kindle, FedEx) Attended (participants were asked to recall products advertised in commercials) No cue validation	M&Ms. (sweet) Timing: during cue exposure Outcome: Grams (weighed)	Portion size: small (200 g), large (400 g) Restraint: low, high (TFEQ [58]) Time of day: 9 am–12 pm, 12 pm–5 pm Sex: males, females Perceived dieting success: low, high Hunger and fullness (pre- and post study; 7-point Likert scale) BMI: low, high M&M liking: low, high Consumption frequency of M&Ms.: low, high	Effect of condition on food intake approached significance with lower food intake in experimental compared to control. The effect of condition on food intake was found only in restrained eaters in the large portion size condition, not small portion size. Unrestrained eaters’ food intake did not significantly differ between experimental and control conditions in small and large portions conditions.	Assumed random assignment based on study 1; No procedures to control for appetite between conditions (hunger and fullness at pre-cue exposure did not differ between conditions); Cover story used and participants who guessed the study aims were excluded; Of the 224 participants, 59 did not consume any test foods Means and SDs used in meta-analysis were for males’ and females’ intake obtained from author For subgroup restraint analysis, means, SDs and sample sizes were estimated No test of mechanism
Werle (2017); Pilot study; FR [44]	Laboratory; between-subjects; random assignment to conditions; Student sample, *n* = 95 (48.4% males – based on correspondence with author) (*n* = 46 in prime condition); Age: 20.2 (0.8); BMI: 21.2 (2.7) (self-reported); 17 excluded (details not specified).	Commercials (duration: 30 s) Experimental: featured men and women engaged in sports (e.g. rugby, running; Nike); Control: featured peacocks (Telus telecommunications). Attended (participants rated advert on multiple attributes)	M&Ms. (sweet) Timing: Immediately after cue exposure Outcome: grams (weighed)	Sex: males, females	No significant differences in food intake between conditions (effect of condition approached significance, *p* = .09) Gender x condition interaction on food intake was non-significant.	No procedures to control for appetite between conditions; Cover story used, no info about participants’ beliefs about study; No test of mechanism

^a^Sample size (n) refers to remaining sample size after exclusions removed; For age (years) and BMI (kg/m^2^) values show mean (SD) unless stated

^b^In the meta-analysis, morning and afternoon sessions were treated as two separate studies; Article also reported mean food intake in response to an ‘indulgent’ condition; means for healthy and control were used in our analysis only

^c^Article also reported mean food intake in response to a tempting (eating enjoyment) preload (garlic bread); means for diet-congruent and control were used in our analyses only

^d^Article also reported mean food intake in resposne to a snacking (2 fast-food products, candy bar, and cola soft drink) message advert; means for nutrition message and control were used in our analyses only

^e^Article also reported a different manipulation for male participants (*n* = 151) which was not included in our analyses; Article included four conditions: control; weight control images only; weight control cue with congruent weight control text and weight control cue with incongruent text. In the analyses the overall effects of primes are reported compared to control adjusting for sample size accordingly

^f^Article also reported means for a ‘fat models’ condition, means for ‘thin models’ and ‘neutral slides’ were included in our analyses only

^g^Article also reported means for a ‘large bodies’ condition, means for ‘thin bodies’ and ‘product only’ were included in our analyses only

^h^Article also reported means for a ‘gourmet’ condition, means for ‘dieting’ and ‘control’ were included in our analyses only

^i^Small and large packs were used as two separate studies in the analyses

Twelve studies used female participants only [12, 13, 18, 20–23, 27–30] (including the study where only the manipulation for females was deemed eligible [32]) and 14 used mixed-sex samples [9–11, 14–16, 19, 26, 40–44]. Of the mixed-sex samples, 42.6% were males. Eighteen studies used student samples [10, 14, 15, 18, 19, 21, 22, 26–30, 32, 40–42, 44], three used non-student samples [9, 16, 43] and five used a combination of student and non-student samples [11–13, 20, 23]. Mean age was 24.8 ± SD 9.5 years (median 20.3; range 18.6–56.0 years) (mean age unavailable for two studies [19, 30]). Mean BMI from available studies (*n* = 17 [10, 12, 13, 15, 16, 21–23, 26–29, 32, 40, 42, 44]) was 23.4 ± SD 1.5 kg/m^2^ (median 23.0; range 21.2–26.5 kg/m^2^). This was based on nine studies where participants’ self-reported BMI [10, 15, 16, 21, 29, 32, 40, 42, 44] and six where BMI was objectively measured by the researcher [12, 13, 22, 23, 27, 28]. Methods for obtaining BMI were not specified in two studies [26].

### Other study information

All studies examined the effect of cues on short term food intake; however, there were variations in the interval between cue exposure and assessment of food intake. Thirteen studies assessed food intake either during cue exposure (*n* = 3 [9, 15, 29]) or immediately after cue exposure (*n* = 10 [10–12, 14, 19, 26, 30, 42, 44]), including those that also administered appetite ratings after cue exposure. For one study this was after a second exposure phase to counteract a lexical decision task [12]. In one study timing differed for each participant [16] and in another study a lexical decision task was administered after cue exposure for half of the participants while food intake of the other half was assessed immediately after cue exposure [18]. The remaining 11 studies administered tasks in between cue exposure and food intake which consisted of a lexical decision task [13, 23, 28], cognitive load [43] and self-control task [40], rating the cue exposure task and completing a demographic questionnaire [41], a self-concept questionnaire [32], a filler memory task implicit mood task and weight satisfaction [22], a self-esteem task only^2^ or with either a mood and image forced choice recognition task [27] or a mood and body size perception task [21]. Based on the questionnaires or tasks used, the four studies using tasks to assess mood, body satisfaction or self-esteem were classified in the meta-analysis as studies inducing negative body image or mood.

The majority of studies assessed snack intake; except for two which examined either lunch [42] or evening meal intake [13]. Of the studies providing snack foods, nine provided a sweet food: cookies [18, 21], M&Ms. [14, 15, 22, 44], chocolate [9], raisins [26] and chocolate or blueberries [11]; five studies provided savoury snacks: pretzels [32], crisps [41, 43], crackers and pretzels [30] and meat samples [16]. Nine provided a selection of sweet and savoury foods [10, 12, 19, 20, 23, 26, 28, 29, 40]. One study used a high calorie food that had been individually selected [27]. Twelve studies reported gram intake [10, 11, 15, 18–22, 28, 30, 43, 44]; 10 reported energy intake [12, 13, 23, 26, 27, 29, 40–42]; three reported piece count [9, 16, 32] and one reported intake in ounces [14]. Most studies (*n* = 21) examined at least one moderating variable in response to cue exposure (see Table 2).

A range of weight control cues were used across studies (see Table 2). Eleven studies used thin models [9, 11, 18, 20–22, 27, 28, 30, 32, 43], five used low calorie foods [12, 13, 15, 19, 23], five used exercise cues [26, 40, 42, 44], three used a combination of healthy, exercise [10] and body weight or shape cues (e.g. word thin) [14, 41], one used a weight loss television programme [29] and one used a poster that referred to a low calorie recipe and slim fig. [16]. In most studies, participants attended to the cue (*n* = 18 [10, 13–15, 19–23, 26, 28–30, 32, 40–42, 44]). To engage attention, participants were either asked to rate the cue on various attributes (e.g. if presented as an advert or image) [21, 26, 32, 42, 44], encode the cue for subsequent recall [15, 20, 22, 28, 30], eat or smell and handle the cue [13, 23], form sentences, create sentences or complete a word search task containing cue-relevant words [10, 14, 41], watch a television programme containing cues [19, 29] or the researcher directed participants’ attention to the cue [40]. Five studies used incidental exposure (achieved with slim figures on a computer screensaver [9, 11, 43], having magazines that featured slim models in the testing rooms [18] and a poster on the window of a butcher’s store [16]) and three used subliminal exposure (15–23 millisecond exposure to exercise words [26] and images of slim models [27] or low calorie food and beverages [12]). In eight studies the weight control cues were selected based on either pre-tests which validated that the cues were associated with dieting to lose weight [13, 23] or health and nutrition [19]; others validated body images as being slim and attractive as rated by either pre-study samples [27, 32] or researchers [21]; and other studies obtained ratings from the study participants that the cues were associated with dieting to lose weight [12] or made them feel ‘healthy’ and ‘in shape’ [42].

In terms of explaining the effects of exposure to weight control cues on food intake, seven studies assessed potential mechanisms; four studies administered tasks to assess the accessibility of diet-related goals (goal priming) [12, 13, 18, 23] and three assessed self-esteem [20, 27], mood [21, 27] or body size perception [21] as potential mechanisms for cues influencing food intake.

### Meta-analysis

**Fig. 2:**
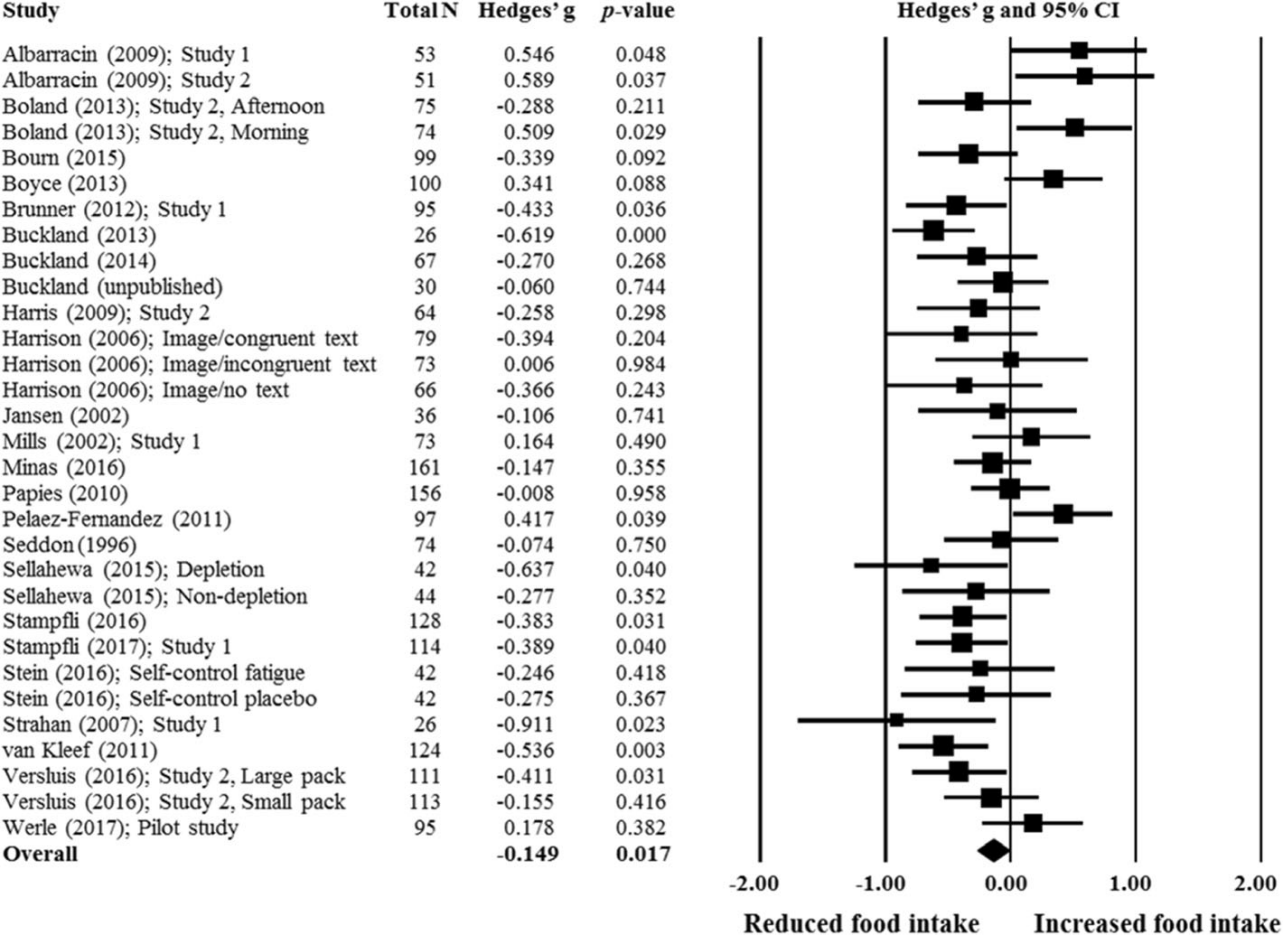
Forest plot of comparisons between exposure to weight control and control cues on food intake

There was a trivial overall mean effect size of cue exposure in reducing food intake (ES: -0.149, 95% CI: -0.271 to − 0.027; *n* = 31; Fig. 2), which was statistically significant from zero (*p* = 0.017). Heterogeneity among the studies was moderate (I^2^ = 56.88%). Sensitivity analysis based on the one-study-removed procedure did not reveal any major impact of a single study on the overall effect size.

#### Planned moderator analysis: Cue type and level of engagement

Results from the moderator analyses are presented in Table 3. Cue type and level of engagement did not significantly impact the variation in the effect of cue exposure on food intake. However, there were effect sizes significantly different from zero favouring a reduction in food intake when cues were low calorie foods or thin models, and when cues were attended to. Important to note, there was low variability across studies using low calorie foods (I^2^ = 20.47%).

**Table 3:** Subgroup and moderator analyses

Moderator variables	Subgroup level	*p* for between subgroup heterogeneity	Effect size (Hedges’ g) (95% CI)^i^
*Subgroups*			
WC goals	Weak WC goals (*n* = 13)	0.351	−0.071 (− 0.329, 0.187)
	Strong WC goals (*n* = 13)		−0.248 (− 0.517, 0.020)
WC goals (removing negative affect^ii^)	Weak WC goals (*n* = 10)	0.020	0.014 (−0.249, 0.278)
	Strong WC goals (*n* = 10)		−0.440 (− 0.718, − 0.163)**
Sex	Females (*n* = 5)	0.056	−0.305 (− 0.574, − 0.036)*
	Males (*n* = 5)		0.057 (− 0.200, 0.314)
*Categorical moderators*			
Cue type	Exercise (*n* = 6)	0.309	0.018 (−0.268, 0.303)
	Low kcal foods (n = 6)		−0.302 (− 0.560, − 0.044)*
	Mixed (*n* = 5)		−0.098 (− 0.403, 0.206)
	Thin models (*n* = 9)		−0.249 (− 0.476, − 0.022)*
	Thin models – negative (*n* = 4)		0.105 (−0.238, 0.448)
	TV show (*n* = 1)		−0.339 (− 0.968, 0.290)
Cue engagement	Attended (*n* = 23)	0.616	−0.169 (− 0.316, − 0.023)*
	Incidental (*n* = 5)		−0.160 (− 0.444, 0.124)
	Subliminal (*n* = 3)		0.061 (−0.375, 0.498)
Cue validated	Not validated (*n* = 21)	0.213	−0.098 (− 0.242, 0.046)
	Validated (*n* = 10)		−0.263 (− 0.478, − 0.047)*
Sample type	General community (*n* = 3)	0.363	−0.263 (− 0.616, 0.090)
	Mixed (*n* = 5)		−0.295 (− 0.579, − 0.010)*
	Students (*n* = 23)		−0.091 (− 0.236, 0.055)
Sex	Females (*n* = 13)	0.884	−0.137 (− 0.333, 0.059)
	Mixed (*n* = 18)		−0.156 (− 0.316, 0.004)
Cue-food intake interval^iii^	During/immediately (*n* = 16)	0.091	−0.188 (− 0.347, − 0.030)*
	After tasks (*n* = 9)		−0.284 (− 0.496, − 0.072)**
	After negative tasks (*n* = 4)		0.107 (− 0.216, 0.431)
	Counterbalanced (*n* = 1)		0.417 (−0.173, 1.006)
	Varied (*n* = 1)		−0.008 (− 0.545, 0.528)
Snack type	Not reported (*n* = 1)	0.132	−0.106 (− 0.894, 0.681)
	Savoury (*n* = 8)		−0.316 (− 0.549, − 0.083)**
	Sweet (*n* = 11)		0.023 (−0.167, 0.213)
	Sweet and savoury (*n* = 11)		−0.221 (− 0.426, − 0.017)*
Outcome	Energy intake (*n* = 11)	0.849	−0.167 (− 0.380, 0.046)
	Grams (*n* = 12)		−0.136 (− 0.338, 0.067)
	Grams z-scores (*n* = 1)		−0.258 (− 0.984, 0.467)
	Ounces (*n* = 2)		0.108 (−0.390, 0.606)
	Piece count (*n* = 5)		−0.227 (− 0.554, 0.101)
Controlled appetite^iv^	No control (*n* = 15)	0.016	−0.008 (− 0.169, 0.154)
	Controlled for (*n* = 16)		−0.289 (− 0.451, − 0.128)***
Theoretical approach^v^	Body image (*n* = 9)	0.427	− 0.125 (− 0.350, 0.099)
	Goal priming (*n* = 19)		−0.231 (− 0.367, − 0.096)**

*Note*. ^i^Effect size, 95% confidence intervals and asterisks denoting statistical significance refer to the subgroup level; ^ii^Post-hoc analyses; ^iii^Interval between cue exposure and assessment of food intake; ^iv^Based on either study procedures or including reported appetite in analyses; ^v^Two studies using motor priming were not included in the moderator analysis as both were from one article [26], another using vicarious goal fulfilment was also not included [46]. *p<.05; **p<.01; ***p<.001 at the subgroup level

#### Planned subgroup analysis: Weight control goals

Subgroup analysis on data reported in 13 studies (contributing 26 effect sizes) showed no significant variation in the effect of cue exposure on food intake between groups with weak or strong weight control goals (Table 3). However, as heterogeneity was moderate in the strong weight control goal group (I^2^ = 65.11%) [low heterogeneity in the weak weight control goal group (I^2^ = 0.00%)] and there was concern that some studies were confounded with negative affect after cue exposure, exploratory subgroup analysis was conducted with these studies removed [20, 21, 27]. Upon removal, the analysis showed significant variation in the impact of cue exposure between subgroups. For participants with strong weight control goals, cue exposure decreased food intake compared to control with a small-to-moderate effect size, which differed significantly from zero (heterogeneity slightly decreased: I^2^ = 56.84%), whereas for participants with weak weight control goals, the effect of cue exposure on food intake was trivial and non-significant. In two of these studies (contributing 3 effect sizes) [15, 16] means and standard deviations had to be estimated by the research team (see Table 2). Removal of these studies did reduce the effect of the moderator to non-significant (*p* = 0.159); however, the effect size for participants with strong weight control goals remained significant and small-to-moderate (ES: -0.378, 95% CI: -0.733 to − 0.023; *p* = 0.037, *n* = 7).

#### Exploratory analyses

Table 3 shows the moderators examined. The only moderator with a significant impact on the variation of the effect of cue exposure on food intake was whether appetite had been controlled for. Food intake significantly decreased after exposure to weight control cues relative to control and with small-to-moderate effects in studies that controlled for appetite (either using study procedures or including appetite ratings in the analysis). The impact of sex on the effect of cue exposure on food intake approached significance (*p* = .06), with food intake significantly decreasing after exposure to weight control cues with a small effect size in females.

It is also of interest to note that, within the other moderators, significant effect sizes favouring a reduction in food intake with cue exposure were apparent with validated cues, in mixed student-community samples, in studies that assessed food intake during or immediately after cue exposure or after tasks that did not induce negative affect, savoury snacks, combined sweet and savoury snacks, and in studies that used a goal priming theoretical approach.

#### Risk of bias

**Fig. 3:**
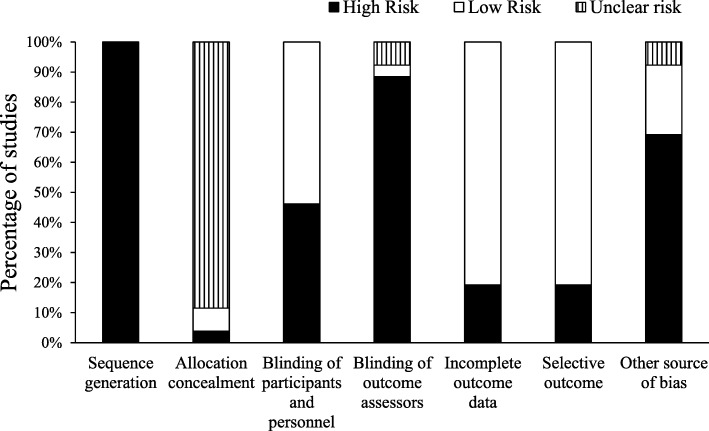
Risk of bias summary

A summary for risk of bias is shown in Fig. 3. For ‘sequence generation’, all studies were rated at high risk; two did not randomly assign participants to conditions [16, 43], three studies did not specify whether randomisation had been used or randomisation had to be assumed based on a previous study reported in the article [9, 11, 15]; all remaining studies specified random allocation to conditions or random order of conditions (within-subject designs [13, 23]) but no studies reported randomisation methods used and as such were rated at high risk.

No studies specified whether allocation concealment had been used. As such all studies were rated as unclear risk, except for those using a repeated measures design (low risk) [13, 23] and a study conducted by one of the current authors who confirmed allocation concealment had not been used [12]. For the criteria ‘blinding of participants and personnel’, twelve studies were rated at high risk for either not providing information about a cover story or providing a cover story but not reporting whether participants believed the cover story [9, 18, 20, 26, 29, 32, 40–44]. All other studies were rated at low risk for blinding of participants and personnel. For blinding of outcome assesors, all studies were rated at high risk, except for two that were unclear [19, 34] and one that was rated as low risk [43]. For the criteria ‘incomplete outcome data’ four studies were considered at high risk for excluding participants for reasons not given in the exclusion criteria [22, 28, 42, 43]. For the criteria ‘selective outcomes,’ five studies were considered to be at high risk; two did not report means and standard deviations for non-significant results (planned analyses) [40, 41], two studies reported unplanned analyses [11, 43] and one study had conducted two additional separate conditions but due to non-significant findings merged the data from these conditions with the weight control and control conditions [42]. For ‘other bias’, eighteen studies were considered at high risk for a variety of confounding variables (some studies had multiple confounding variables): using piece count to assess intake (*n* = 5 [9, 16, 26, 32], not using procedures to control for subjective appetite (e.g. fasting period, fixed meals or including subjective appetite ratings or duration since last ate as a covariate, *n* = 11 [11, 14, 16, 18, 21, 26, 29, 32, 41, 43]); assessing intake in the presence of social others (*n* = 3 [16, 32, 42]), administering psychometric scales prior to assessing food intake (e.g. weight satisfaction, self-esteem, body size perception; *n* = 4 [20, 21, 27, 28]), providing a restricted portion of food (n = 4 [9, 11, 26, 43]) and not measuring all foods provided (n = 1 [42]). Two studies were rated as unclear for either not specifying the control procedure [40] (e.g. for the experimental condition the researcher diverted participants’ attention to cues, no information provided about the procedure for the control condition) or not specifying whether food intake was assessed individually or in the presence of social others [19]. The remaining six studies were rated at low risk for ‘other bias’ [10, 12, 13, 22, 23, 30].

### Publication bias

Inspection of the funnel plot (Additional file 3) showed a slight shift to the left of the mean, suggesting some presence of publication bias. Egger’s regression intercept revealed little evidence of publication bias (intercept: 0.33, 95% CI: -2.15 to 2.81, *p* = 0.788); however, the trim-and-fill analysis revealed evidence of four missing studies reporting increased food intake in response to cue exposure to bring symmetry to the right of the mean. These studies, would have to have an ES ≥ 0.4, to moderate the ES to − 0.080 (95% CI -0.208 to 0.047), negating its significance.

## Discussion

This systematic review and meta-analysis assessed the effect of exposure to weight control cues on food intake. Results from the meta-analysis, which combined 24 articles (25 studies) and contributed 31 effect sizes, suggest that in general, exposure to weight control cues has a trivial effect to reduce food intake compared to control cues. The magnitude of this effect was increased in individuals with strong weight control goals (identified as being either dieters, restrained eaters or those with high-self discrepancy). Cue type and level of engagement with cues did not moderate the effect. However, the effect sizes were more consistent (as indicated by low heterogeneity) and significant when low calorie foods were used as the weight control cue. The effect sizes were also significant for thin models (with no negative affect) and when participants attended to the weight control cues. Studies using incidental and subliminal cue exposure did not significantly affect food intake.

To our knowledge this is the first systematic review and meta-analysis examining the effect of weight control cues on food intake. The findings support narrative reviews that weight control cues can reduce food intake, especially in individuals with strong weight control goals [8, 17]. This finding is also in accordance with the Goal Conflict Theory which states that cues will elicit a greater response in those with relevant goals [45]. In the current meta-analysis, this selective response to weight control cues (based on the strength of weight control goals) suggests the results might be due to goal priming. However, further investigation is required to confirm goal priming as a mechanism. Previous research has shown that exposure to weight control cues increases accessibility of weight control goals [46]. In this meta-analysis, there were insufficient studies which tested goal priming as a mechanism to be able to draw clear conclusions. As such, it is recommended that studies investigating the effects of weight control cues on food intake incorporate tests to identify possible goal priming mechanisms. One of the reasons why few studies incorporated tests of goal priming mechanisms may be because the tests themselves can disrupt or confound the effects of cue exposure on subsequent food intake. Thus, researchers need to identify effective methods to overcome this issue such as counterbalancing the order that the mechanism and food intake is assessed [18], repeating cue exposure after testing the mechanism [12, 23] or devising alternative tasks based on the principles of goal priming [45].

The Goal Conflict Theory proposes that weight control cues will have selective effects on those with strong weight control goals because such individuals hold conflicting weight control and eating enjoyment goals [8]. As both eating enjoyment and weight control goals cannot be active at the same time, activation of one will lead to inhibition of the other. For example, in food-tempting settings, the eating enjoyment goal becomes more prominent and the weight control goal is momentarily inhibited, resulting in behaviour consistent with eating enjoyment goals. In contrast, weight control cues reinstate the weight control goal and facilitate controlled food intake in tempting food environments. Individuals with weak weight control goals do not experience such conflict and therefore their behaviour is less determined by environmental cues. Thus, the selective response to weight control cues found in this meta-analysis is consistent with the Goal Conflict Theory.

Yet, caution is needed when interpreting the finding that the strength of weight control goals moderated the effect of weight control cues on food intake, as this effect was only found after removing the studies which induced negative affect after cue exposure (by administering scale assessing weight satisfaction, self-esteem, body size discrepancy and negative mood scales). These differences in methodologies might have partly explained the moderate heterogeneity observed and justified removing these studies. Indeed, the difference that removing these studies made to the overall effect is consistent with a recent review which suggested that weight control cues will be most effective if they are associated with positive affect [17]. It is possible that studies that induce awareness of body image after cue exposure activate alternative processes which undermine the effects of cue exposure. However, it should be acknowledged that there were a number of studies which examined weight control goals as moderators but the means and standard deviations could not be obtained [11, 19, 22, 40–42]. Thus, further research comparing the effect of weight control cues in individuals with strong and weak weight control goals is needed. Direct comparisons between exposure to weight control cues only and exposure to weight control cues with tasks that increase awareness of body image are also needed. Furthermore, this meta-analysis assessed the impact of weight control cues as moderated by weak and strong weight control goals. Yet, very few studies included samples who were engaged in an active weight control attempt [12, 13, 23] most used measures of restrained eating to determine weight control goals [11, 15, 16, 18, 19, 40–42]. It has been argued that restrained eating assesses the tendency to watch what one eats rather than engaging in weight control strategies per se [47]. Thus, the effect of weight control cues on food intake need to be evidenced in more samples who are actively engaged in weight control attempts. It is also important to note that the grouping of low and high weight control goals in this meta-analysis was an exploratory analysis.

It is interesting that level of engagement with the cue did not significantly moderate the effects of weight control cues on food intake. However, this may be an issue of power as only a small number of studies investigated incidental and subliminal cues. It is important to note that attending to cue exposure did have a significant effect in reducing food intake and this finding is consistent with literature on mindfulness. Mindful eating involves focusing on the sensational experience of eating and food-related thoughts and it has been shown to reduce cravings [16, 48] and food intake [49]. This meta-analysis suggests that focusing on a weight control cue can also decrease food intake in those with strong weight control goals.

Controlling for appetite was another methodological difference between studies. Exploratory analyses showed effect sizes were larger in studies which controlled for appetite compared to those that did not. Using procedures to control for appetite (such as participants fasting for a given period of time or being provided with a fixed-caloric meal) reduces non-systematic variance in food intake and improves the quality of the research design. Although not able to test here, it is possible that appetite moderates the effect of cue exposure on food intake and therefore it is important to control for it. Based on this finding we would strongly recommend researchers adopt standardised procedures when conducting laboratory studies [24, 38]. Adoption of such procedures is also important as the quality assessment showed that most studies were rated as at high risk of bias (Additional file 2). This recommendation is in line with a recent call for future laboratory eating behaviour studies to adopt more rigorous methods [50].

The current findings have important implications. While future studies are needed to confirm the durability of the effects of weight control on food intake (e.g. after repeated exposure), exposure to weight control cues has relevance for weight control attempts. Of course, it cannot be assumed that short term reductions in food intake will result in long term weight changes [51, 52]. As such, the impact of weight control cues on food intake and changes in body weight over time needs to be tested. Subsequent research could test the effects of incorporating weight control cues into a weight loss programme on changes in body weight. In today’s technology-rich environment, weight control cues could be delivered via smart phone applications and used with ease and minimal cost [53].

Consideration of the limitations of the systematic review and meta-analysis is needed. To our knowledge, there are no validated databases of weight control cues and as such, the research team used their judgement about which cues qualified as being weight control cues. For instance, the selection criteria used did not include eating enjoyment cues as weight control cues (e.g. palatable food or overweight models [54]). However, it has been suggested that for some individuals, eating enjoyment cues themselves might activate weight control cognitions [55]. For example, individuals who report high levels of dieting success may have learned over time to associate eating enjoyment cues with weight control cognitions, meaning that weight control goals are activated in response to eating enjoyment cues [55]. As this may involve different processes to more ‘prototypical’ weight control cues, eating enjoyment cues were not included in the current meta-analysis. Importantly, this issue highlights that across studies there may be subjectivity in the definition and selection of cues used to activate weight control cognitions. It is possible that some studies may have used cues that the sample did not associate with weight control (even if the researchers assumed they did) and thus, this may have minimised the opportunity to observe effects of weight control cues in some studies. In support of this, the current findings showed that effects were only significant in studies that validated cues either before or during the study as being associated with weight control constructs (although not a significant moderator). Therefore, it will be valuable for future research to develop a validated database of weight control cues that report the extent to which a range of cues are associated with weight control and the extent to which these vary within and between sub-populations (for example, age, restrained eaters, types of dieters - successful versus less successful weight losers and maintainers). This will be a valuable resource for researchers to use when conducting goal priming studies.

The current findings are also limited to the data available. There were a number of studies which examined weight control goals [11, 19, 22, 40–42] or other moderators such as BMI [40, 42] and exercise levels [42] that were not included in the meta-analysis due to insufficient data being available. Additionally, the risk of bias assessment showed that due to methodological issues, most of the studies were rated as being at high risk of bias and as such the findings should be interpreted with caution. Moreover, in terms of public health applications, the current findings provide support that weight control cues can improve acute control over food intake in individuals with goals to lose weight. This support is based predominantly on laboratory-based findings and as such more studies in real world settings are needed before applying such strategies to public health initiatives. This is important because although only small-to-moderate effects were reported for individuals with strong weight control goals, when scaled up and integrated as part of a wider national-level strategy tackling overconsumption, such effects can have an important impact on the population [56]. However, weight control cues will likely to have no impact on individuals who do not have weight control goals, who might also be the target of public health behaviour change interventions. As such, the findings suggest that alternative methods are needed that consider individual motivations or goals.

## Conclusion

Results from this systematic review and meta-analysis showed weight control cues can reduce food intake and more so in individuals with strong weight control goals. However, the effects of weight control cues in those with strong weight control goals were only apparent when studies increasing body image awareness (and thus negative affect) were removed, suggesting that to impact food intake weight control cues should be presented in the absence of negative affect. The mechanisms underlying this effect remain to be evidenced and further studies are required to confirm which types of cues and level of engagement are most effective.

## Additional files

Additional file 1: Electronic Supplementary Information Detailed search strategy (example database search). Description: keys terms used in the electronic database search. (DOCX 18 kb).

Additional file 2: Risk of bias assessment. Description: Table showing risk of bias assessment for each study (DOCX 19 kb).

Additional file 3: Risk of bias funnel plot. Description: Figure showing risk of bias funnel plot (DOCX 50 kb).

## Footnotes

1 For instance, in three studies snacks were provided seven [14 (Study 1) confirmed in correspondence with author], fourteen [62] and twenty minutes [63] before cue exposure.

2 Seddon and Berry (1996) and Mills, Polivy, Herman & Tiggemann (2002) used The State Self-esteem Scale [64]; Jansen & de Vries (2002) participants rated state depression and state satisfaction on visual analogue scales.
