# Supplementary material for: Priming food intake with weight control cues: systematic review with a meta-analysis
Source: Int J Behav Nutr Phys Act. 2018 Jul 9;15:66. doi: 10.1186/s12966-018-0698-9 (PMC6038287; doi:10.1186/s12966-018-0698-9)
Supplement: Supplementary file 1 — Electronic Supplementary Information Detailed search strategy (example database search). Description: keys terms used in the electronic database search. (DOCX 18 kb). [file 12966_2018_698_MOESM1_ESM.docx]

Database: PsycINFO <1806 to January Week 3 2017>

1 Prime.mp.

2 Priming.mp.

3 Primed.mp.

4 Exposure.mp.

5 Cue$.mp.

6 Reminder.mp.

7 Goal activation.mp.

8 Images.mp.

9 weight loss.mp. or Weight Loss/

10 diet.mp. or Diet/

11 Health goals.mp.

12 Slim.mp.

13 Weight control.mp.

14 Energy intake.mp. or Energy Intake/

15 Calori* intake.mp.

16 food intake.mp.

17 food consumption.mp.

18 eating behavio*.mp.

19 1 or 2 or 3 or 4 or 5 or 6 or 7 or 8 (236807)

20 9 or 10 or 11 or 12 or 13 (32998)

21 14 or 15 or 16 or 17 or 18 (31110)

22 19 and 20 and 21 (540)

23 limit 22 to (english language and humans and ("adolescent (13 to 18 years)" or "young adult and adult (19-24 and 19-44)" or "middle age (45 to 64 years).
