# Supplementary material for: Priming food intake with weight control cues: systematic review with a meta-analysis
Source: Int J Behav Nutr Phys Act. 2018 Jul 9;15:66. doi: 10.1186/s12966-018-0698-9 (PMC6038287; doi:10.1186/s12966-018-0698-9)
Supplement: Supplementary file 2 — Risk of bias assessment. Description: Table showing risk of bias assessment for each study (DOCX 19 kb). [file 12966_2018_698_MOESM2_ESM.docx]

| First author (year) | Sequence generation | Allocation concealment | Blinding of participants and personnel^i^ | Blinding of outcome assessors^ii^ | Incomplete outcome data^iii^ | Selective outcome | Other source of bias^iv^ |
| --- | --- | --- | --- | --- | --- | --- | --- |
| Experimental studies | | | | | | | |
| Albarracin (2009); Study 1 | High risk | Unclear risk | High risk | High risk | Low risk | Low risk | High risk |
| Albarracin (2009); Study 2 | High risk | Unclear risk | High risk | High risk | Low risk | Low risk | High risk |
| Boland (2013); Study 2 | High risk | Unclear risk | Low risk | High risk | Low risk | Low risk | High risk |
| Bourn (2015) | High risk | Unclear risk | High risk | High risk | Low risk | Low risk | High risk |
| Boyce (2013) | High risk | Unclear risk | Low risk | High risk | High risk | Low risk | Low risk |
| Boyce (2014) | High risk | Unclear risk | Low risk | High risk | High risk | Low risk | High risk |
| Brunner (2012); Study 1 | High risk | Unclear risk | High risk | High risk | Low risk | Low risk | High risk |
| Buckland (2013) | High risk | Low risk | Low risk | High risk | Low risk | Low risk | Low risk |
| Buckland (2014) | High risk | High risk^v^ | Low risk | High risk | Low risk | Low risk | Low risk |
| Buckland (unpublished) | High risk | Low risk | Low risk | High risk | Low risk | Low risk | Low risk |
| Harris (2009) | High risk | Unclear risk | Low risk | Unclear risk | Low risk | Low risk | Unclear risk |
| Harrison (2006) | High risk | Unclear risk | High risk | Unclear risk | Low risk | Low risk | High risk |
| Jansen (2002) | High risk | Unclear risk | Low risk | High risk | Low risk | Low risk | High risk |
| Mills (2002); Study 1 | High risk | Unclear risk | Low risk | High risk | Low risk | Low risk | High risk |
| Minas (2016) | High risk | Unclear risk | High risk | Low risk | Low risk | High risk | High risk |
| Papies (2010) | High risk | Unclear risk | Low risk | High risk | Low risk | Low risk | High risk |
| Pelaez-Fernandez (2011) | High risk | Unclear risk | High risk | High risk | Low risk | Low risk | High risk |
| Seddon & Berry (1996) | High risk | Unclear risk | High risk | High risk | Low risk | Low risk | High risk |
| Sellahewa (2015) | High risk | Unclear risk | Low risk | High risk | Low risk | Low risk | Low risk |
| Stampfli (2016) | High risk | Unclear risk | High risk | High risk | High risk | High risk | High risk |
| Stampfli (2017); Study 1 | High risk | Unclear risk | Low risk | High risk | Low risk | High risk | High risk |
| Stein (2016) | High risk | Unclear risk | High risk | High risk | Low risk | High risk | Unclear risk |
| Strahan (2007); Study 1 | High risk | Unclear risk | Low risk | High risk | Low risk | Low risk | Low risk |
| van Kleef (2011) | High risk | Unclear risk | High risk | High risk | High risk | High risk | High risk |
| Versluis (2016); Study 2 | High risk | Unclear risk | Low risk | High risk | Low risk | Low risk | High risk |
| Werle (2017); Pilot study | High risk | Unclear risk | High risk | High risk | High risk | Low risk | High risk |

^i^Based on whether a cover story was used and believed by participants.

^ii^Based on whether the experimenter who assessed food intake was blind to the study aims or condition administered.

^iii^Based on whether the exclusion of participants was specified in the exclusion criteria or exclusions deviated from standard procedures in the research field

^iv^Based on risk of confounding variables influencing food intake [e.g. used piece count (susceptible to researcher bias), absence of procedures to control for appetite between conditions; social test settings; administering psychometric scales before assessing food intake].

^v^Not reported in the paper but this study was conducted by the lead author of the meta-analysis (NB).
