# Supplementary figures and images for: Priming food intake with weight control cues: systematic review with a meta-analysis
Source: Int J Behav Nutr Phys Act. 2018 Jul 9;15:66. doi: 10.1186/s12966-018-0698-9 (PMC6038287; doi:10.1186/s12966-018-0698-9)

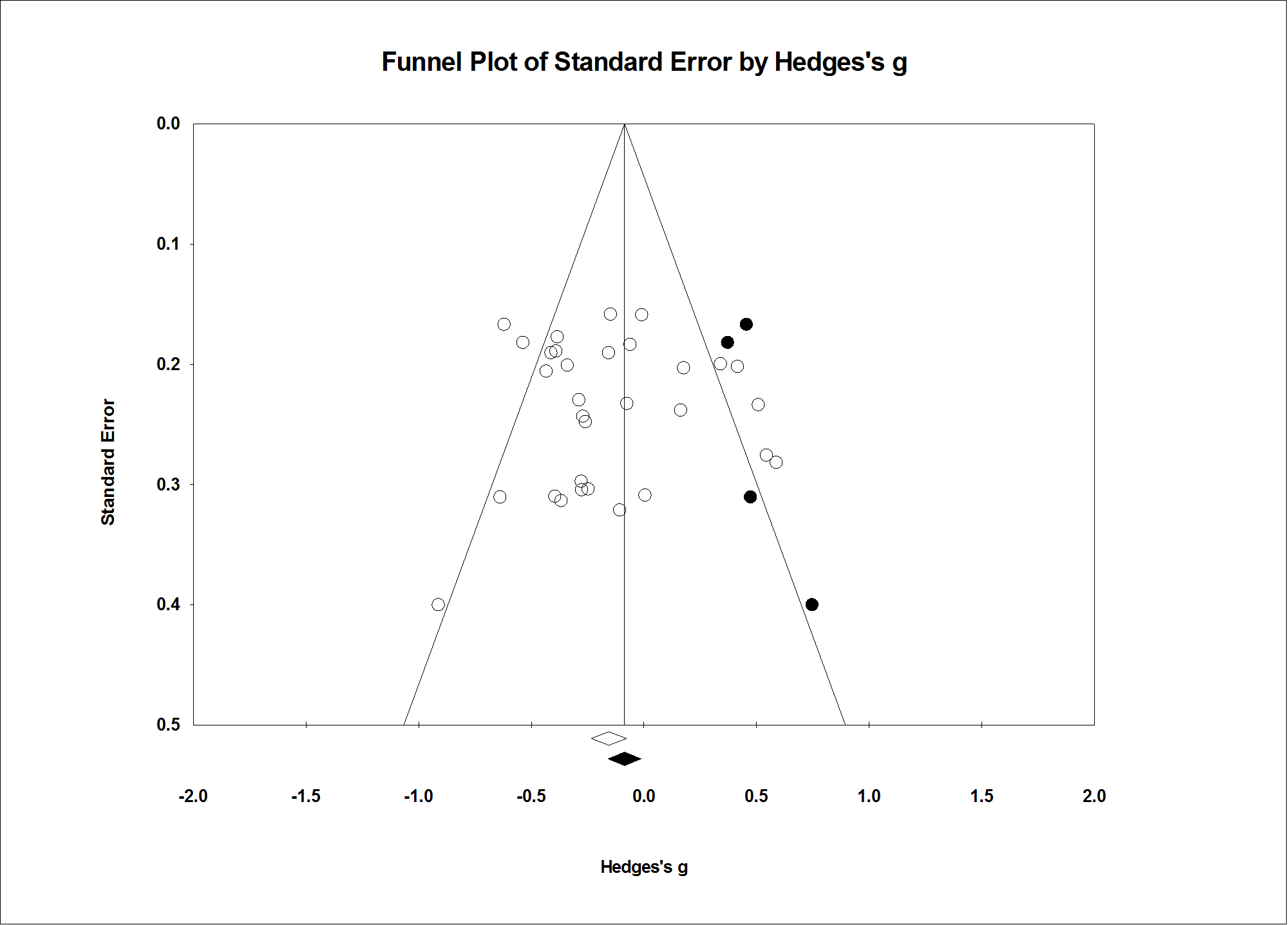

Supplement: Supplementary file 3 — Risk of bias funnel plot. Description: Figure showing risk of bias funnel plot (DOCX 50 kb). [file 12966_2018_698_MOESM3_ESM.docx]
